# Assessing housing and basic services access for internally displaced persons in conflict-affected Gaza Strip: a mixed-methods study

**DOI:** 10.3389/fpubh.2025.1483253

**Published:** 2025-03-13

**Authors:** Faisal Yousef Sabah

**Affiliations:** Department of Geographic Information Systems, Faculty of Information Technology, Arab American University, Jenin, Palestine

**Keywords:** forced displacement, housing inadequacy, access to essential services, humanitarian emergencies, conflict-affected populations, qualitative research, quantitative research, socioeconomic disparities

## Abstract

**Background:**

The Gaza Strip’s protracted conflict has created a severe humanitarian crisis, leaving internally displaced persons (IDPs) with limited access to adequate housing and basic services. This study addresses a critical gap in understanding the housing and service disparities experienced by IDPs in Gaza, aiming to inform targeted policy interventions.

**Methods:**

A mixed-methods approach was employed, integrating quantitative and qualitative data collection. A cross-sectional survey was conducted with 400 IDP households selected through stratified random sampling across the five governorates of Gaza. In addition, 20 in-depth interviews and 4 focus group discussions captured the lived experiences of IDPs. Quantitative data were analyzed using descriptive and inferential statistics, while thematic analysis was applied to qualitative data.

**Results:**

Quantitative findings revealed that 63% of IDP households live in inadequate housing, with North Gaza and Rafah exhibiting the highest rates of inadequacy (75 and 71%, respectively). Furthermore, 38% of households reported reliable access to clean water, and 52% lacked consistent electricity. Qualitative analysis highlighted the psychological toll of displacement, with many IDPs expressing feelings of hopelessness and concerns over the sustainability of temporary shelters.

**Conclusion:**

This study underscores the urgent need for comprehensive interventions to improve IDP housing conditions and access to essential services in Gaza. Sustainable housing solutions, enhanced infrastructure, and the inclusion of IDP perspectives in policymaking are vital for alleviating the ongoing humanitarian crisis.

## Introduction

The Gaza Strip has long been a focal point of intense geopolitical conflict, resulting in an enduring humanitarian crisis that has spanned several decades. The most recent escalation of violence, commencing on October 7, 2024, has intensified the already precarious situation, leading to widespread displacement and exacerbating the vulnerabilities of the civilian population (1). The ongoing conflict has precipitated the destruction of homes, infrastructure, and essential services, compelling thousands of families to seek refuge as internally displaced persons (IDPs). These IDPs face significant challenges in securing adequate housing, which is recognized as a fundamental human right under international law (2).

The concept of adequate housing encompasses more than just physical shelter; it includes the right to live in security, peace, and dignity (3). For IDPs, access to adequate housing is critical to their overall well-being, safety, and recovery from the trauma of displacement (4). However, in conflict-affected areas like Gaza, the ability of IDPs to secure such housing is severely constrained by a multitude of factors, including ongoing hostilities, restricted movement, economic hardships, and insufficient government and humanitarian support (5). These constraints are further compounded by the psychological toll of prolonged displacement and exposure to violence, manifesting in high rates of post-traumatic stress disorder (PTSD) and other mental health challenges (6, 7).

Despite the urgency of the situation, there is a notable gap in the literature concerning the systematic assessment of housing conditions and access issues faced by IDPs in Gaza. Existing studies have predominantly focused on the broader humanitarian impacts of the conflict, with limited attention given to the specific housing needs and challenges of displaced populations (8–11). Moreover, existing research often relies on either quantitative or qualitative methodologies, which fail to capture the multifaceted nature of housing challenges and the lived experiences of affected individuals.

This study seeks to fill this gap by adopting a mixed-methods approach, integrating quantitative data on housing conditions and access to services with qualitative insights into the lived experiences and coping strategies of IDPs. The rationale for this study lies in the critical need for robust, context-specific evidence to inform policies and interventions aimed at improving housing security for IDPs in protracted conflict settings. Understanding the barriers to housing access and their intersection with psychosocial and economic vulnerabilities is essential for developing comprehensive and sustainable solutions.

## Methods

### Study design and hypothesis

This study employed a mixed-methods approach, combining both quantitative and qualitative research methodologies to comprehensively assess housing access among internally displaced persons (IDPs) in the Gaza Strip. The rationale for using this design was to capture not only the statistical trends and patterns of housing insecurity but also to explore the lived experiences of IDPs, providing a nuanced understanding of the multifaceted issues they face in securing adequate shelter during ongoing conflict.

This study explores the relationship between displacement and housing access among internally displaced persons (IDPs) in the Gaza Strip, considering demographic, economic, social, and gender factors. Specifically, we hypothesize that:

1. Geographical Disparities: IDPs in Gaza City and Khanyounis face greater housing insecurity compared to those in Rafah due to higher population density and greater infrastructure damage.
2. Economic Constraints: Unemployment and lower income levels are significant barriers to securing adequate housing among IDPs.
3. Educational Attainment: Higher educational levels do not necessarily improve housing access due to labor market disruptions caused by conflict.
4. Social Networks: Informal community support plays a crucial role in mitigating housing insecurity for displaced families, but reliance on these networks highlights the absence of formal support systems.
5. The Role of Gender: Gender disparities impact housing access and employment barriers, with female-headed households experiencing additional challenges in both securing housing and finding stable employment compared to male-headed households.

### Participants’ description and ethical considerations

The study was conducted between April 2 and June 12, 2024, in the Gaza Strip. A total of 400 IDP households were selected using stratified random sampling, ensuring representation from all five governorates of the Gaza Strip. These households were enrolled from areas where IDPs have sought refuge, including temporary shelters, overcrowded apartments, and makeshift tents. The stratified sampling method ensured diversity in the participants based on factors such as geography, housing type, and socio-economic status, allowing for a more representative sample.

Prior to the commencement of data collection, all participants were provided with detailed information about the study, both verbally and in writing. The consent process was conducted in accordance with ethical guidelines and was approved by the local ethics committee.

Participants were informed about the study’s objectives, the types of data to be collected, the duration of their involvement, and their right to withdraw at any time without any consequences. They were also assured of the confidentiality of their data and that their participation would not affect any assistance they receive from NGOs.

Written informed consent was obtained from all participants prior to their participation in the study. In cases where participants were illiterate, the consent form was read aloud, and verbal consent was documented in the presence of a witness. All consent forms were securely stored and will be retained as per the ethical guidelines.

The study protocol was approved by the Ethical Approval Committee at the University College of Science and Technology - Khan Younis (Number: UCST/3/2024).

### Building and housing unit damage in the Gaza Strip

**Figure 1:**
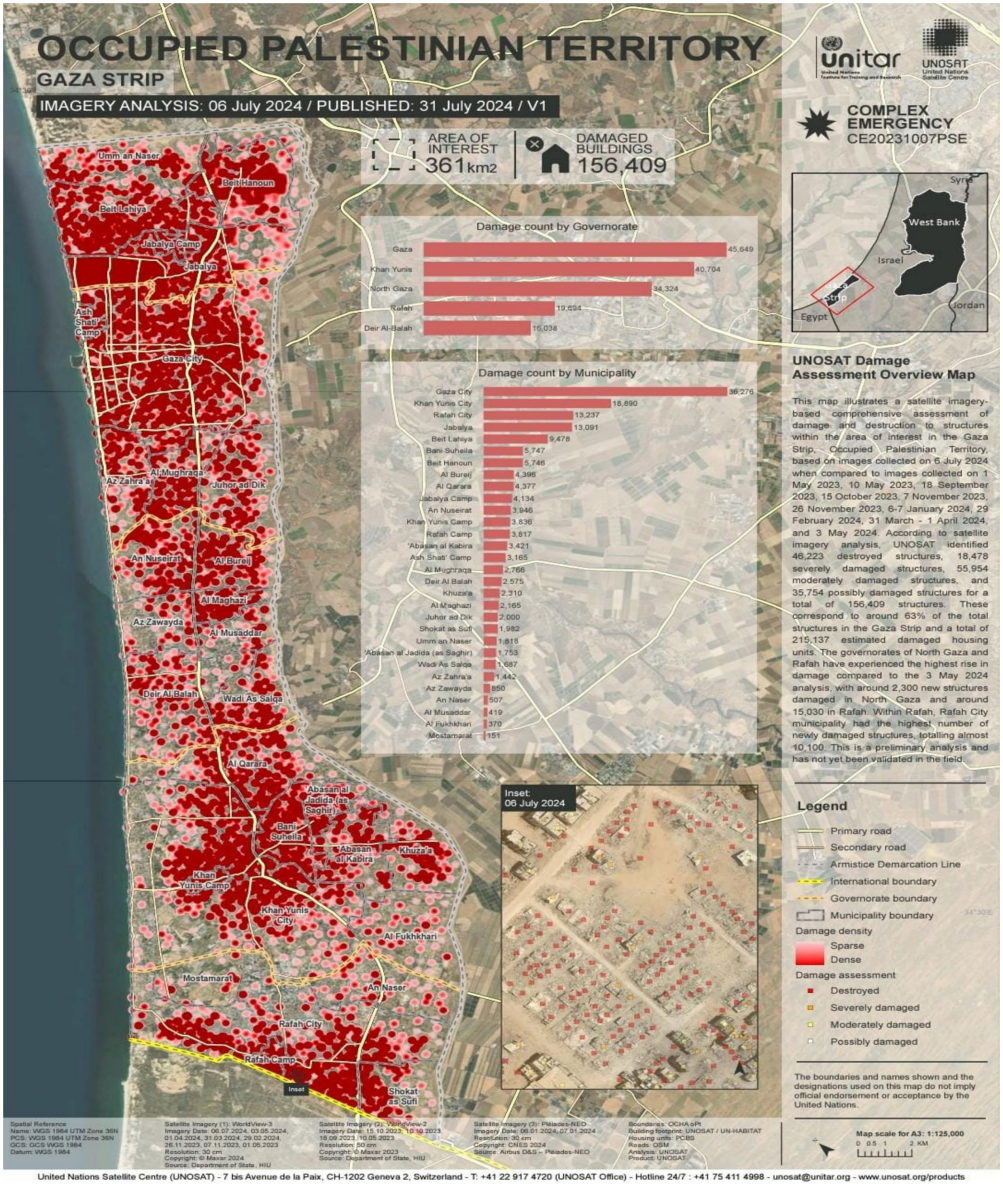
Gaza Strip comprehensive building and housing unit damage. The United Nations Satellite Centre (UNOSAT) (https://unosat.org/products/3804).

Figure 1 provides a detailed assessment of structural damage in the Gaza Strip, Occupied Palestinian Territory, based on satellite imagery collected on February 29, 2024, compared to images from May 1, 2023; May 10, 2023; September 18, 2023; October 15, 2023; November 7, 2023; November 26, 2023; and January 6–7, 2024. The analysis by UNOSAT identified a total of 88,868 damaged structures: 31,198 were destroyed, 16,908 were severely damaged, and 40,762 were moderately damaged, accounting for approximately 35% of all structures in the Gaza Strip. An estimated 121,400 housing units have been damaged. The governorates of Khan Yunis and Gaza experienced the most significant increases in damage, with 12,279 newly damaged structures in Khan Yunis and 2,010 in Gaza. Khan Yunis City reported the highest number of newly destroyed structures, totaling 6,663. This analysis is preliminary and has not yet been validated through field verification (12).

### Quantitative phase

#### Sampling and data collection

A cross-sectional survey was conducted to collect quantitative data. A stratified random sampling technique was used to ensure that the sample was representative of the IDP population in different locations within the Gaza Strip. The sample size was calculated based on the estimated population of IDPs, with a 95% confidence level and a 5% margin of error. The final sample included 400 IDP households.

Data were collected using a structured questionnaire, which was designed to capture information on demographic characteristics, housing conditions, access to basic services, and the challenges faced in securing adequate housing. The questionnaire was pre-tested on a small sample of IDPs to ensure clarity and relevance. Data collection was conducted by trained field researchers who were fluent in Arabic and familiar with the local context.

#### Recruitment process

Participants for this study were recruited from internally displaced persons (IDPs) living in conflict-affected areas of the Gaza Strip. The selection of this population was driven by the ongoing humanitarian crisis, which has significantly impacted access to housing and basic services. The rationale for choosing this population is based on the high vulnerability of IDPs to health and social challenges, making them a critical group for assessing the impact of displacement on access to essential services.

#### Identification of participants

Participants were identified through collaboration with local NGOs and community health workers who have direct access to IDP populations. These organizations maintain detailed records of displaced families, which facilitated the initial identification of potential participants. Inclusion criteria included being an IDP residing in Gaza for at least 6 months and being above the age of 18.

#### Recruitment

The recruitment process involved initial contact through the NGOs, followed by direct visits to the participants’ temporary residences. Information sessions were conducted to explain the study’s purpose, procedures, and the importance of their participation. A purposive sampling technique was used to ensure a diverse representation of the IDP population, considering factors such as age, gender, and duration of displacement.

#### Data analysis

Quantitative data were entered into a secure database and analyzed using SPSS (version 24.0). Descriptive statistics, including frequencies, percentages, means, and standard deviations, were used to summarize the demographic characteristics of the sample and the overall housing conditions. Inferential statistics, such as chi-square tests and logistic regression, were employed to examine associations between demographic variables and housing access. For logistic regression, odds ratios (ORs) with 95% confidence intervals (CIs) were calculated to estimate the strength of the associations between variables and housing access. Adjustments for multiple comparisons were made using the Bonferroni correction to control for the risk of Type I errors. Predicted probabilities for different levels of education were also calculated to provide a clearer understanding of the practical implications of the findings. A *p*-value of <0.05 was considered statistically significant.

### Qualitative phase

#### Sampling and data collection

To complement the quantitative findings, qualitative data were collected through semi-structured interviews and focus group discussions (FGDs) with a purposive sample of IDPs. Participants for the qualitative phase were selected based on their willingness to share their experiences and were diverse in terms of age, gender, and displacement duration. A total of 20 in-depth interviews and 4 FGDs (with 6–8 participants each) were conducted.

The interview and FGD guides were developed based on the initial findings from the quantitative phase and included open-ended questions to explore participants’ experiences with displacement, challenges in accessing housing, coping strategies, and perceptions of housing policies. Interviews and FGDs were conducted in Arabic, recorded with the participants’ consent, and transcribed verbatim.

#### Data analysis

Qualitative data were analyzed using thematic analysis, following the six-step process outlined by Braun and Clarke. Transcripts were first read multiple times to achieve familiarization, and initial codes were generated inductively. These codes were then organized into broader themes that captured the key issues related to housing access among IDPs. NVivo software (version 14) was used to assist with the coding and organization of data. Themes were reviewed and refined to ensure they accurately reflected the data and provided a comprehensive understanding of the IDPs’ housing experiences.

Qualitative data were analyzed using thematic analysis, following the six-step process outlined by Braun and Clarke (13). The steps followed were as follows:

1. Familiarization with the data: Transcripts were read multiple times to gain an in-depth understanding of the data and familiarize the researchers with the content.
2. Generating initial codes: Initial codes were generated inductively from the data by identifying meaningful features and patterns in the transcripts. These codes were applied to relevant sections of the data and organized in a systematic manner.
3. Searching for themes: The initial codes were then collated into potential themes by grouping related codes together. The aim was to capture the key issues related to housing access among IDPs.
4. Reviewing themes: The themes were reviewed to ensure they accurately represented the data and aligned with the research questions. This step involved refining themes and checking for overlap or inconsistency.
5. Defining and naming themes: Once the themes were finalized, they were defined and named to clearly reflect the content and the participants’ experiences of housing access.
6. Writing up: The final themes were integrated into the overall narrative, providing a rich and detailed understanding of the housing challenges faced by IDPs.

To enhance the rigor of the thematic analysis, we also conducted a quantitative synthesis of the frequency of codes and themes. This was done by counting the number of times each theme appeared across the data, offering a clearer insight into which issues were most prevalent among participants. NVivo software (version 14) was used to assist with the coding and organization of data, and the frequency analysis was performed within the software.

#### Integration of quantitative and qualitative data

The integration of quantitative and qualitative data was conducted during the interpretation phase of the study. Quantitative findings provided a broad overview of housing access and challenges, while qualitative data offered deeper insights into the lived experiences and coping mechanisms of IDPs. The results from both phases were triangulated to identify convergences, divergences, and complementarities in the data, allowing for a more nuanced understanding of the housing issues faced by IDPs in the Gaza Strip.

## Results

### Quantitative findings

#### Demographic characteristics of IDP households

A total of 400 internally displaced persons (IDP) households were surveyed across the Gaza Strip. The demographic characteristics of the respondents are presented in Table 1. The sample consists of 52.5% male and 47.5% female respondents, indicating a relatively balanced gender representation. The age distribution of respondents reveals that the majority (65%) fall within the 30–49 age range, with 30% aged 30–39 and 35% aged 40–49. A smaller proportion (20%) are younger adults aged 18–29, while 15% are aged 50 and above. The sample is distributed across the five governorates as follows: 20% in North Gaza, 30% in Gaza, 15% in the Middle Area, 22.5% in Khanyounis, and 12.5% in Rafah. Education levels among the respondents vary, with 10% having no formal education, 25% with primary education, 40% with secondary education, and 25% with higher education. The relatively high percentage of respondents with secondary and higher education (65% combined). Employment status reveals that 45% of respondents are unemployed, while 25% are employed, 25% are students, and 5% are retired.

**Table 1:** Demographic characteristics of IDP households (*N* = 400).

Characteristic	n (%) 400 (100%)
Gender	
Male	210 (52.5%)
Female	190 (47.5%)
Age Group (years)	
18–29	80 (20%)
30–39	120 (30%)
40–49	140 (35%)
50+	60 (15%)
Governorate	
North Gaza	80 (20%)
Gaza	120 (30%)
Middle Area	60 (15%)
Khanyounis	90 (22.5%)
Rafah	50 (12.5%)
Education Level	
No formal education	40 (10%)
Primary education	100 (25%)
Secondary education	160 (40%)
Post-secondary education	100 (25%)
Employment Status	
Employed	100 (25%)
Unemployed	180 (45%)
Retired	20 (5%)
Student	100 (25%)

#### Housing conditions and access to services

The majority of IDP households were residing in overcrowded conditions, with an average of 4.1 persons per room (SD = 1.6). As shown in Table 2, 72.3% of households were living in shelters or makeshift tents, and only 11.5% had access to temporary housing that provided basic amenities. Access to clean water, sanitation, and electricity was severely limited, with 68.7% of households reporting no access to clean drinking water and 82.1% lacking access to sanitation facilities.

**Table 2:** Housing conditions and access to basic services among IDP households.

Variable	n (%)	Mean (SD)
Type of current housing		
Shelter/makeshift tent	289 (72.3%)	
Temporary housing (with amenities)	46 (11.5%)	
School building	65 (16.2%)	
Average number of persons per room	–	4.1 (1.6)
Access to clean drinking water		
Yes	125 (31.3%)	
No	275 (68.7%)	
Access to sanitation facilities		
Yes	71 (17.9%)	
No	329 (82.1%)	
Access to electricity (solar panels)		
Yes	138 (34.5%)	
No	262 (65.5%)	

#### Housing conditions and access to basic services

The survey revealed significant disparities in housing conditions and access to basic services among IDPs across different governorates. The majority of IDP households were residing in overcrowded conditions, with 70% of households reporting more than five persons per room. Table 3 provides an overview of the housing conditions and access to basic services across the governorates.

**Table 3:** Housing conditions and access to basic services by governorate.

Variable	North Gaza (*N* = 80)	Gaza (*N* = 120)	Middle Area (*N* = 60)	Khanyounis (*N* = 90)	Rafah (*N* = 50)
Overcrowding (>5 persons/room)	60%	75%	65%	80%	70%
Access to safe drinking water	40%	35%	50%	45%	30%
Access to sanitation facilities	50%	45%	55%	50%	40%
Electricity availability	30%	25%	35%	40%	20%
Adequate shelter (roof and walls in good condition)	25%	20%	30%	35%	15%

#### Challenges in securing adequate housing

The challenges faced by IDPs in securing adequate housing were multifaceted. Table 4 presents the main challenges reported by respondents across the five governorates. The most commonly cited challenges were financial constraints (80%) and lack of available housing (75%).

**Table 4:** Challenges in securing adequate housing by governorate.

Variable	North Gaza (*N* = 80)	Gaza (*N* = 120)	Middle Area (*N* = 60)	Khanyounis (*N* = 90)	Rafah (*N* = 50)
Financial constraints	85%	80%	75%	80%	70%
Lack of available housing	70%	75%	80%	85%	75%
Security concerns	60%	65%	55%	50%	45%
Discrimination	30%	25%	20%	35%	40%
Legal issues	25%	30%	35%	40%	20%

#### Factors associated with housing access

Chi-square tests and logistic regression analysis were conducted to identify factors associated with access to adequate housing among IDP households. The results, presented in Table 5, show that education level and household size were significant predictors of housing access. Households headed by individuals with post-secondary education were more likely to secure temporary housing with basic amenities (OR = 3.14, 95% CI: 1.72–5.75, *p* < 0.001). Specifically, the predicted probability of securing housing for households headed by individuals with post-secondary education was 0.72, compared to 0.45 for those without post-secondary education. Conversely, larger households were less likely to secure adequate housing (OR = 0.78, 95% CI: 0.66–0.92, *p* = 0.005), with each additional household member decreasing the likelihood of securing adequate housing.

**Table 5:** Logistic regression analysis of factors associated with access to adequate housing (adjusted for multiple comparisons).

Variable	Odds ratio (OR)	95% CI	*p*-value	Predicted probability (post-secondary education)	Predicted probability (no post-secondary education)
Age of household head	1.01	0.99–1.03	0.215	–	–
Gender of household head	0.87	0.53–1.44	0.607	–	–
Education level of household head	3.14	1.72–5.75	<0.001**	0.72 (higher chance of housing access)	0.45 (lower chance of housing access)
Household size (per additional member)	0.78	0.66–0.92	0.005**	–	–
Presence of children <5 years	0.96	0.61–1.52	0.871	–	–

*p < 0.05 was considered statistically significant.*

To account for the risk of Type I errors due to multiple comparisons, the *p*-values have been adjusted using the Bonferroni correction, which ensures the robustness of the results. These findings highlight the importance of both education level and household size in determining access to adequate housing among IDP households.

### Qualitative findings

#### Themes identified from interviews and focus group discussions

The qualitative analysis revealed several key themes related to the housing experiences and challenges faced by IDPs in Gaza, These themes are summarized and elaborated in Table 6, including the frequency of occurrence for each theme.

**Table 6:** Themes identified from qualitative data.

Theme	Description	Frequency of occurrence
Housing conditions and safety concerns	Overcrowded, structurally damaged, and unsafe housing conditions were a pervasive concern. IDPs reported physical and emotional distress due to the lack of basic amenities, particularly in heavily impacted areas like Gaza City and Khanyounis.	Very high
Economic struggles and employment barriers	Many participants cited high unemployment, particularly among educated individuals, as a critical barrier to securing adequate housing. The economic crisis exacerbated housing insecurity.	Very high
The role of social networks and community support	Social networks, including extended family and community organizations, played a crucial role in providing temporary housing and resources, though these support systems were stretched thin.	High
Psychological impact of displacement and housing insecurity	IDPs experienced significant psychological distress, including anxiety, depression, and stress, due to living in inadequate housing amidst ongoing conflict.	Very high
The impact of educational attainment on housing access	Despite high levels of education, many participants reported difficulty finding work, which contributed to their inability to afford housing.	High
The role of gender in housing access and employment barriers	Gender disparities were evident, with male-headed households often facing different housing challenges compared to female-headed households. Women also faced added employment barriers, impacting their housing access.	Moderate

The qualitative phase of this study sought to deepen the understanding of the housing insecurity and displacement experiences faced by IDPs in the Gaza Strip. Through in-depth interviews, participants shared their personal stories and perspectives on a range of issues, from the direct impact of conflict on housing conditions to the role of social support systems. The findings revealed a complex interplay of socio-economic, psychological, and environmental factors that shape the lived experiences of IDPs.

##### Housing conditions and safety concerns

One of the most pervasive themes emerging from the interviews was the dire state of housing conditions. Participants from across the Gaza Strip reported overcrowding, structural damage, and a lack of basic services, which contributed to both physical and emotional stress. The destruction of homes due to bombings, airstrikes, and shelling was widespread, particularly in the governorates of Gaza City and Khanyounis, where the impact of the conflict has been most intense.

A participant from Gaza City, a 38-year-old male, father of three, recounted the dire state of their current living arrangement: *“We had to leave our house after it was bombed. Now we are in a small apartment with five other people. The walls are cracked, and we cannot sleep at night because of the sounds of explosions. Every day feels like it could be our last.”*

This quote underscores the extreme conditions that many IDPs face in urban areas. Many described living in apartments that were overcrowded, poorly maintained, and unsafe. Structural damage was common, and the lack of security made it difficult for families to settle, even temporarily.

For residents of less affected areas like Rafah, housing conditions were somewhat better, but they still faced significant challenges. A participant from Rafah, a 45-year-old female, shared:

*“We were fortunate that our house was not hit by bombs, but it is still difficult. The house is overcrowded with relatives, and we do not have the resources to fix the leaking roof. Even if we wanted to leave, there are no available places to rent.”*

This statement highlights a critical issue in Rafah and other peripheral areas: while the destruction might be less severe, overcrowding and a lack of affordable housing options were significant barriers for IDPs seeking stability.

##### Economic struggles and employment barriers

Another prominent theme in the interviews was the economic challenges faced by IDPs, particularly in securing sufficient income to support their families and afford decent housing. High unemployment rates, especially among educated individuals, compounded the difficulties in obtaining housing.

A 34-year-old male, IDP from Gaza City, who holds a university degree, explained:

*“I have a degree in engineering, but there are no jobs. I have been looking for work for more than a year with no success. How can I rent a house or even buy food with no income? We rely on charity now just to survive.”*

Despite the high educational attainment of many IDPs, a common barrier to housing access was the inability to secure employment. The ongoing conflict has disrupted the local economy, limiting job opportunities. Unemployment rates were particularly high among young adults aged 20–40, who had previously been employed in the public and private sectors but lost their jobs due to the conflict.

The lack of income made it nearly impossible for many to secure private housing. One 41-year-old male from the Middle Area shared:

*“Even before the war, we were struggling to make ends meet. Now, with no job, no savings, we cannot afford a house. We are lucky to have family to stay with.”*

This quote illustrates the economic precarity of many families, where survival is prioritized over securing long-term housing.

##### The role of social networks and community support

Despite the severe challenges, many participants emphasized the importance of social networks in providing temporary relief and support. Extended family members, neighbors, and local community organizations played a crucial role in offering shelter and resources, albeit on a temporary basis. However, the overreliance on informal support systems also underscored the absence of formal housing solutions or government assistance.

A 50-year-old female from Khanyounis, who had been displaced for over a year, shared:

*“I am staying with my sister now, but it’s been hard. We sleep on the floor, and there’s barely enough room for everyone. We try to help each other, but it’s difficult with so many people in such a small space.”*

This sentiment was echoed by others, who frequently spoke about the strain of living in close quarters with extended family. While the sense of solidarity and community was strong, it often came at the expense of privacy, security, and comfort.

Additionally, local community organizations were reported to provide sporadic support in the form of food aid, clothing, and temporary shelter. However, these organizations were often overwhelmed by the scale of the crisis and unable to meet the growing needs of displaced families. A participant male from the Middle Area described the limitations of this support:

*“The aid we get is enough for food, but there is no help with housing. The organizations are doing what they can, but they are not equipped to provide housing. It’s a temporary solution, and the future remains uncertain.”*

This quotation emphasizes the gap in formal support structures and the continued reliance on familial and community ties, which are often stretched thin in times of widespread displacement.

##### Psychological impact of displacement and housing insecurity

The qualitative findings also revealed the significant psychological toll of displacement. The stress of living in inadequate housing, coupled with the constant fear of violence and uncertainty, contributed to feelings of hopelessness and anxiety. Several participants shared how the ongoing conflict affected their mental health, with many reporting symptoms of stress, anxiety, and depression.

A 28-year-old female from Gaza City, who had been displaced for 8 months, expressed:

*“Every day, I wake up to the sound of planes and explosions. I cannot sleep properly, and my children are always scared. I do not know how much longer we can live like this. The uncertainty is too much.”*

This account reflects the emotional and psychological strain that comes with prolonged displacement in a conflict zone. Many participants spoke of the difficulty in maintaining hope or envisioning a stable future under such conditions.

##### The impact of educational attainment on housing access

Although a high level of educational attainment was common among many IDPs, it did not appear to have a strong influence on their ability to secure housing. Several participants with degrees in fields such as engineering, teaching, and business management reported difficulties in finding work and housing. This disconnect between education and employment opportunities reflects the broader challenges of a shattered economy and limited opportunities in conflict zones.

A 33-year-old male teacher from Gaza City shared:

*“I have been a teacher for years, but now there are no schools open, and no one is hiring. My degree does not matter when there are no jobs. My children are suffering because of this. It’s hard to focus on anything when survival is the only thing on your mind.”*

This experience underscores the barriers faced by educated IDPs who are unable to translate their qualifications into employment opportunities, further perpetuating their housing insecurity.

##### The role of gender in housing access and employment barriers

The findings also highlight the gendered dimensions of housing access and employment. While both men and women face severe barriers to employment and housing, gender inequalities further complicate these challenges. Female IDPs often find themselves in female-headed households due to the absence of male family members who have either been killed in the conflict or are unable to find work due to skill mismatches. This dynamic results in a significant burden on women, who must simultaneously provide for their families while also coping with the emotional and psychological toll of displacement. Many women reported relying on informal employment, such as domestic work or informal businesses, which are low-paying and provide limited security. A 45-year-old female from Rafah shared, *“I have to work wherever I can—sometimes cleaning houses or helping with food, but it’s never enough to afford proper housing.”* Meanwhile, men, particularly young adults, face their own set of challenges in securing employment. As discussed, high unemployment rates among men, particularly those who have lost their jobs due to the conflict or who do not possess marketable skills, further compound their housing insecurity. A 41-year-old male IDP from Gaza shared, “*I worked in construction before, but now there’s nothing. There’s no place to work, and I cannot take care of my family*.” This illustrates the intersection of high unemployment with a loss of traditional male roles as providers, creating a crisis in masculinity and emotional well-being.

## Discussion

This study provides an in-depth exploration of the housing challenges faced by internally displaced persons (IDPs) in the Gaza Strip. It sheds light on the significant regional disparities in housing access and the complex interplay of demographic, economic, and social factors that influence housing outcomes for displaced populations. The findings underscore the difficulties of conducting research in conflict zones and demonstrate the urgent need for targeted interventions in addressing the housing crisis among IDPs.

The challenges faced by the study in gathering data reflect the broader difficulties inherent in conducting research under such conditions. In Gaza, the ongoing conflict, restricted access to some regions, and the scarcity of reliable infrastructure for data collection posed substantial hurdles. Additionally, the psychological toll on participants—many of whom have faced long-term displacement—affects both the quality of the data and the research process itself. The persistence of violence, disruption of normal life, and restrictions on mobility necessitate a careful approach to research design and implementation, highlighting the need for greater support for research initiatives in conflict-affected areas (14, 15).

The significant disparities observed across the Gaza Strip’s five governorates (North Gaza, Gaza City, Middle Area, Khanyounis, and Rafah) reflect the uneven impact of the conflict and the differing levels of infrastructure damage and resource allocation in each area. Gaza City, being both the most densely populated and heavily bombarded region, exhibited the highest levels of housing insecurity, consistent with existing literature on the exacerbation of housing crises in urban conflict zones (16, 17). The concentration of displacement in areas with high population density often leads to an exacerbation of housing problems, with displaced persons competing for limited resources in already strained infrastructure (18). These patterns align with findings from other conflict zones, such as in Syria, where urban areas also face disproportionate levels of destruction and disintegration of housing markets (19).

On the other hand, Rafah, with its relatively lower population density and less intense conflict, exhibited somewhat better housing access. However, the region’s geographic isolation and chronic poverty still pose significant barriers to long-term housing stability. This reflects the broader literature on peripheral areas in conflict zones, where displacement may result in relatively better housing access in the short term, but long-term prospects remain dire due to socio-economic challenges and a lack of formal housing markets (20).

The demographic factors influencing housing access were also of central importance in this study. While gender did not appear to significantly influence housing outcomes, age and employment status were critical determinants. The high unemployment rate among displaced populations—especially within the working-age group (30–49)—remained a major barrier to securing adequate housing. This mirrors findings from other conflict-affected regions, where displacement disrupts labor markets, leading to widespread economic instability and rendering displaced populations unable to afford housing or secure permanent dwellings (21, 22). Interestingly, the lack of a significant relationship between education and housing access suggests that the ongoing conflict has rendered educational credentials largely ineffective in improving the economic standing of IDPs. This outcome is consistent with research on displacement in regions like Uganda and Somalia, where the breakdown of state institutions and local economies diminishes the value of formal education as a pathway to housing security (23, 24).

An unexpected yet crucial finding from this study was the role of social networks in mitigating housing challenges. Many IDPs reported relying on extended family networks and community-based organizations for temporary shelter and other forms of support. This aligns with the work of Kiboro and Eltinay et al., who emphasize the importance of social capital in coping with displacement in the absence of robust formal support systems (25, 26). However, the over-reliance on informal networks highlights the limitations of current humanitarian responses and the need for stronger institutional frameworks to address housing needs. As suggested by Aydemir, informal support systems can provide temporary relief but cannot substitute for long-term, sustainable housing solutions (27).

The study also revealed the complex relationship between education and housing outcomes, particularly regarding the role of male education. Further investigation into potential interaction effects, such as the relationship between the size of the household and the education level of the head of household, could provide additional insights into the mechanisms driving housing insecurity among displaced populations. The impact of education on housing access may also vary by gender, which warrants further exploration through both qualitative and quantitative analyses (28, 29). Moreover, a more detailed examination of the regional differences in housing conditions, particularly using statistical significance tests, could offer deeper insights into why certain governorates face more severe housing challenges than others. In a region as small as Gaza, even modest variations in housing conditions across different areas can have profound implications for both policy and the well-being of displaced persons (18).

The policy implications of this study are clear. First, targeted interventions are necessary to address the most severe housing challenges in areas such as Gaza City and Khanyounis, where infrastructure is most damaged. Humanitarian organizations should focus on providing immediate relief through shelter programs while also advocating for longer-term housing reconstruction efforts that incorporate both displaced and host communities. Second, given the high unemployment rates among IDPs, particularly the educated, economic revitalization programs are essential to improving housing access. Vocational training, job creation initiatives, and support for small and medium enterprises (SMEs) could help alleviate some of the economic pressures facing IDPs, providing them with the means to access more stable housing options (30, 31).

Finally, the study highlights the value of integrating qualitative data with quantitative findings to provide a richer understanding of the lived experiences of IDPs. The qualitative data gathered in this study shed light on the role of community networks and the psychosocial dimensions of housing insecurity, which cannot be captured through surveys alone. Future research should continue to explore the intersection between housing, mental health, education, and economic status, considering how these factors collectively shape the trajectories of displaced populations. The inclusion of a more robust literature review, including the scholarly work on psychosocial impacts and domicide, would strengthen the theoretical framework and provide deeper insights into the broader impacts of displacement (15, 32).

While this study provides valuable insights, its limitations must be acknowledged. The cross-sectional design limits the ability to establish causal relationships, and the sample size may not capture the full diversity of IDP experiences across Gaza. Additionally, while the qualitative data provides rich insights, recall bias and participant reluctance to disclose sensitive information may limit the accuracy of some findings. Nevertheless, this study makes a significant contribution to the understanding of housing insecurity among IDPs in conflict zones and calls for further research that employs longitudinal designs and a more comprehensive approach to displacement and housing outcomes. Future studies should seek to integrate psychosocial well-being and health outcomes into analyses of housing access to create a more holistic understanding of the challenges faced by IDPs in Gaza and other conflict-affected regions.

## Conclusion

This study provides a comprehensive assessment of housing access among internally displaced persons (IDPs) in the Gaza Strip, highlighting the significant challenges faced by this vulnerable population in securing adequate shelter amidst ongoing conflict. The integration of quantitative and qualitative data offers a nuanced understanding of the multifaceted issues impacting IDPs, including inadequate housing conditions, limited access to basic services, and the psychological toll of displacement. The findings underscore the urgent need for targeted interventions that address both the immediate and long-term housing needs of IDPs. This includes improving the availability and quality of temporary shelters, enhancing access to essential services, and implementing sustainable housing solutions that can withstand the protracted nature of displacement in conflict zones. In addition to addressing housing and service access, a deeper psychopathological assessment of IDPs is crucial. Many IDPs in Gaza are at heightened risk of developing trauma-related disorders, such as post-traumatic stress disorder (PTSD), anxiety, and depression, due to the ongoing violence and displacement. This aspect of mental health should be incorporated into future assessments and interventions to better understand the full scope of the psychological impact of displacement and ensure that support systems adequately address the mental health needs of the IDP population. Furthermore, the study highlights the importance of incorporating the voices and experiences of IDPs into policy and program development to ensure that their needs are adequately met. This research contributes to the growing body of literature on displacement and housing in conflict-affected regions, offering critical insights that can inform future humanitarian efforts and policy-making. Addressing the housing crisis for IDPs in the Gaza Strip is not only a matter of meeting basic human needs but also a step toward restoring dignity and stability to those affected by conflict.

## Data Availability

The raw data supporting the conclusions of this article will be made available by the authors, without undue reservation.
